# Beyond the Scan: Adapting Multimodal Lung Cancer Screening for Central and Eastern Europe—Overcoming Systemic Barriers and Epidemiological Confounders

**DOI:** 10.3390/medsci14020259

**Published:** 2026-05-18

**Authors:** Rodica Anghel, Antonia-Ruxandra Folea, Vlad-Luca Moga, Cristian Pavel, Diana Troncotă, Matei Celea, Corneliu-Octavian Dumitru, Andreea-Iren Șerban, Liviu Bîlteanu

**Affiliations:** 1 Faculty of General Medicine, Carol Davila University of Medicine and Pharmacy, 8 Eroii Sanitari Street, 050474 Bucharest, Romania; rodica.anghel@umfcd.ro (R.A.); vlad-luca.moga@drd.umfcd.ro (V.-L.M.); cristian-pavel.pavel@drd.umfcd.ro (C.P.); diana.troncota@drd.umfcd.ro (D.T.); liviu.bilteanu@umfcd.ro (L.B.); 2 Oncological Institute “Alexandru Trestioreanu” Bucharest, 252 Soseaua Fundeni, 022328 Bucharest, Romania; mateicelea@gmail.com; 3 Remote Sensing Technology Institute, German Aerospace Center, Münchener Str. 20, 82234 Wessling, Germany; corneliu.dumitru@dlr.de; 4 Department of Preclinical Sciences, Faculty of Veterinary Medicine, University of Agronomic Sciences and Veterinary Medicine, 105 Splaiul Independentei, 050097 Bucharest, Romania; andreea-iren.serban@fmvb.usamv.ro; 5 Faculty of Biology, University of Bucharest, 91-95 Splaiul Independentei, 050095 Bucharest, Romania; 6 Laboratory for Molecular Nanotechnologies, National Institute for Research and Development in Microtechnologies—IMT Bucharest, 126A, Erou Iancu Nicolae Street, 077190 Voluntari, Romania

**Keywords:** low-dose computed tomography, lung cancer screening, structural barriers, Central and Eastern Europe

## Abstract

**Background/Objectives:** Lung cancer remains the leading cause of cancer-related mortality in Central and Eastern Europe (CEE), where late-stage diagnosis, structural healthcare limitations, and regional epidemiological confounders complicate early detection. This review aimed to synthesize the evidence from Romania, Poland, Hungary, and Bulgaria and to outline a context-adapted multimodal screening strategy for CEE settings. **Methods:** A structured review of PubMed-, Scopus-, and Web of Science-indexed literature published from 2010 through 27 December 2025 was performed, focusing on lung cancer epidemiology, screening, implementation barriers, risk stratification, and adjunctive diagnostic approaches in the four selected CEE countries. A total of 297 articles were included. **Results:** The evidence confirms a persistently high burden of late-stage lung cancer across CEE, driven by tobacco exposure, air pollution, radon, comorbidities, diagnostic delays, fragmented registries, workforce shortages, and marked socioeconomic and geographic inequalities. In addition, tuberculosis-related granulomatous lesions and chronic inflammatory lung disease complicate nodule interpretation and reduce screening specificity in parts of the region. Screening experience from Poland and Hungary supports the feasibility of low-dose computed tomography (LDCT) when paired with volumetric assessment and structured follow-up. Risk-prediction models may improve participant selection, while biological triage may help reduce unnecessary invasive procedures, although prospective validation remains limited. **Conclusions:** In CEE, lung cancer screening should be implemented as a multimodal, context-adapted program combining risk-based enrollment, volumetric LDCT, selective biological triage, smoking-cessation support, and centralized multidisciplinary delivery.

## 1. Introduction

Lung cancer remains the leading cause of cancer mortality globally and a major public health burden in Central and Eastern Europe (CEE) [1,2,3,4,5,6,7,8,9,10,11,12,13,14,15]. In Poland, Romania, Hungary, and Bulgaria, late-stage presentation drives high mortality. Lung cancer is the leading cause of cancer death in Bulgaria (18.3%), with 61% diagnosed at stage IV and many at stage III [16]; in Romania, late diagnosis is compounded by limited access and delays [17,18]. In Poland, stages III–IV predominate, with roughly 35% stage IV in pilot networks [19,20,21]. Hungary also reports among the highest incidence and mortality rates in Europe, with persistent late diagnosis [2,22,23].

European data (EUROCARE-5) confirm poor lung cancer survival with marked East–West disparities [6], while GLOBOCAN 2022 attributes high Eastern European mortality to late diagnosis, limited treatment, and smoking [5]. Even resected early-stage non-small-cell lung cancer (NSCLC) shows an approximately 50% 5-year survival in CEE cohorts [12]. High smoking prevalence and treatment limitations sustain excess mortality, with a clear North-West–South-East gradient across Europe [10,11]. Projections warn that without expanded early detection, disparities will widen, and aging plus tobacco trends will increase absolute mortality, especially in less developed regions [4,24].

Low-dose CT (LDCT) is the standard for secondary prevention, reducing mortality by 20% [National Lung Screening Trial (NLST)] and 24–33% (NELSON trial) [25,26,27]. However, direct transfer to CEE is limited by cost, CT/radiologist shortages, and system fragmentation [28,29,30]. Additionally, the high prevalence of tuberculosis (TB) sequelae in Romania and neighboring regions causes granulomatous lesions mimicking malignancy, complicating nodule assessment and increasing invasive procedures [31,32,33]. Economic constraints impose stricter cost-effectiveness thresholds vs. Western systems [2].

An adapted multimodal strategy is required: LDCT integrated with biological and mathematical tools. Risk models (e.g., PLCOm2012) outperform standard criteria such as those of the National Comprehensive Cancer Network (NCCN) in Poland, reducing unnecessary screening without missing cancers [34]. Circulating biomarkers (metabolomics, microRNA) may decrease false positives from inflammatory lesions [35,36]. Molecular testing may be useful in preventing unnecessary diagnostic workup or procedures [37]. This review synthesizes region-specific evidence on LDCT screening, biomarker research, and risk-prediction models from Romania, Poland, Hungary, and Bulgaria, with the aim of contextualizing current detection strategies, resource allocation challenges, and clinical outcomes within CEE healthcare systems.

The primary objective of this review is to provide a comprehensive and critical overview of lung cancer screening, risk stratification approaches, and emerging biological tools in CEE, with particular focus on Romania, Poland, Hungary, and Bulgaria, in light of the structural and epidemiological characteristics of the region. To support this overarching goal, the study pursues two secondary objectives: first, to analyze the unique country-level epidemiological landscapes and entrenched systemic barriers hindering screening adherence, such as workforce shortages and major diagnostic confounders like tuberculosis; and second, to evaluate how programmatic adaptations, specifically personalized risk stratification and non-invasive serum biomarkers, can safely optimize tumor detection, improve resource allocation, and reduce the burden of unnecessary invasive procedures in these resource-constrained healthcare systems.

While the individual utility of LDCT volumetry, risk-prediction models (e.g., PLCOm2012), and biomarker triage has been demonstrated in isolated studies [34,35], the unique contribution of this review is the synthesis of these distinct methodologies into a unified, multimodal framework tailored specifically for CEE. Current international screening guidelines largely rely on data from high-resource Western populations (such as the US NLST or Dutch–Belgian NELSON trials), which cannot be directly transplanted to CEE settings due to distinct systemic barriers, stricter cost-effectiveness thresholds, and unique epidemiological confounders like endemic tuberculosis [10,28,29]. This review transcends mere aggregation of existing findings by proposing a cohesive implementation strategy that bridges the gap between Western clinical trial efficacy and real-world CEE healthcare realities.

The rest of this review is organized as follows. Section 2 outlines the review methodology and study selection process. Section 3 analyses the epidemiological, clinical, molecular, and risk-factor profiles of lung cancer in Romania, Poland, Hungary, and Bulgaria. Section 4 shifts from barriers to solutions by examining the structural, geographic, socioeconomic, and psychosocial challenges to screening implementation in CEE, while also reviewing multimodal optimization strategies, economic and infrastructural considerations, and country-specific implementation priorities. Section 5 presents the conclusions and future directions.

## 2. Materials and Methods

The structural and epidemiological similarities of CEE healthcare systems prompt this review to focus on lung cancer epidemiology and screening studies from Romania, Poland, Bulgaria, and Hungary. These insurance-based countries spend similar amounts on public health but struggle to implement population-based lung cancer screening.

We clustered evidence from countries with similar health system organization and screening implementation stages to improve contextual comparability and reduce structural confounding from Western European systems with different funding and program maturity.

**Figure 1:**
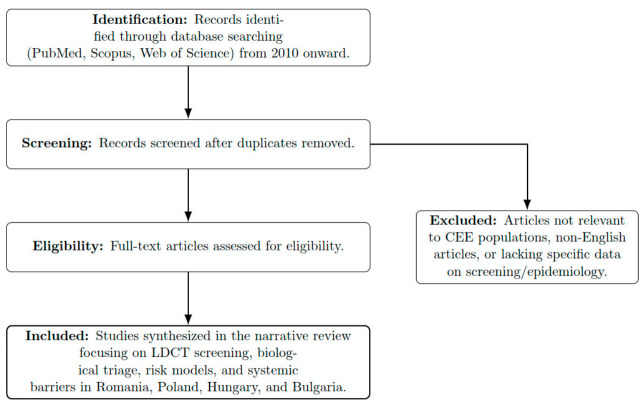
Flowchart of the literature search and study selection process, focusing on lung cancer epidemiology, screening developments, and systemic barriers in Romania, Poland, Hungary, and Bulgaria.

Thus, the eligibility of PubMed-, Scopus-, and Web of Science-indexed studies from these countries from 2010 was assessed (Figure 1). Original research and reviews dominated this analysis.

The search was conducted by two independent reviewers on the 27th of December 2025, and as such, this is the cutoff date for publication. The search formula is presented below:

Lung cancer AND (screening OR treatment OR epidemiology) AND [COUNTRY NAME], with the ‘country name’ tag substituted for each of the four countries included in the analysis (Romania, Poland, Bulgaria, and Hungary).

From the records identified through this search strategy, we additionally selected studies that included our countries of interest within broader comparative analyses (e.g., cross-EU comparisons or multi-country studies encompassing these nations).

**Figure 2:**
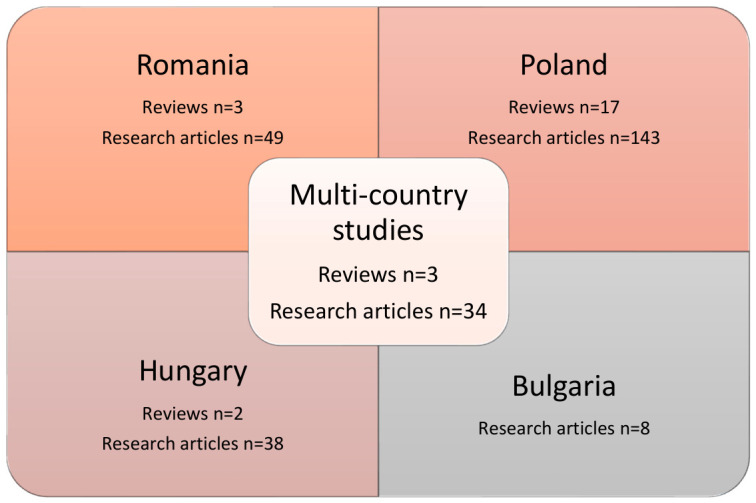
Distribution of included studies across countries, divided into research articles and reviews.

Therefore, after excluding non-English papers, conference abstracts, and papers that did not fit the topic of this analysis, we have included 297 articles in this review (see Figure 2).

## 3. Region-Specific Lung Cancer Landscape

### 3.1. Romania

#### 3.1.1. Epidemiology and Demographics

Romania’s leading cancer killer, especially in men, is lung cancer [17,38,39]. Romania had 11,716 new bronchopulmonary cancer cases (11.2% of all tumor cases) and over 10,000 deaths in 2022, with a cumulative death risk of 3.3% to 3.45% [17,40].

Since there is no national lung cancer registry, regional records are used to estimate national epidemiology, which is unclear despite the high burden [40]. The highest incidence rates per 100,000 people are in southern counties like Giurgiu, and the lowest in Maramureș and Vâlcea [17]. While lung cancer mortality declined or stabilized in many Western countries, it has steadily increased among middle-aged and elderly Romanians [39].

Lung cancer disproportionately affects men. Women have 1511 new cases per year, while men have 6016 [17]. But the gender gap is shrinking. A 5-year Mureș County study found that the male-to-female ratio dropped from 4:1 before the COVID-19 pandemic to 2:1 during it, indicating a steeper decline in male diagnoses and possibly an increase in female tobacco consumption [41,42].

The COVID-19 pandemic caused significant epidemiological fluctuations. In Mureș County, pandemic restrictions led to a 72% drop in new lung cancer diagnoses in the first year, followed by a subsequent rebound [41]. In contrast, Timiș County lung cancer admissions rose 80.5% (2022–2024). Acute CT imaging for COVID-19 respiratory symptoms skyrocketed due to delayed diagnoses, population aging, and accidental tumor detection [40,43,44,45,46]. Romania’s COVID-19 pandemic delayed oncologic treatment by over four weeks and increased mortality rates among SARS-CoV-2-infected patients during chemotherapy or radiotherapy [46].

#### 3.1.2. Clinical, Histopathological, and Molecular Features

Late diagnosis dominates lung cancer in Romania: around 43% stage III and 50–68% stage IV, leading to predominantly palliative care and poor 5-year survival [18,32,46,47,48,49,50,51,52,53,54,55,56,57,58]. Furthermore, the lack of nationwide screening programs delays detection, though pilot projects and adherence to European screening directives are beginning to take shape [17].

Because lung cancer is typically detected at such advanced stages in Romania, it frequently results in widespread and highly atypical metastatic dissemination, such as rare anal or perianal tumors or choroidal metastases that further complicate clinical management and severely affect patient quality of life [48,55]. Moreover, clinicians frequently manage severe local complications, such as malignant pleuropericarditis, where minimally invasive surgical approaches like the thoracoscopic pleuropericardial window are utilized to alleviate symptoms and manage mortality risks [51].

One study showed that diabetic patients from the Dolj region present with advanced stage IV disease much more frequently and exhibit drastically shorter overall survival times compared to non-diabetic lung cancer cohorts [54].

Early detection and surgical resection significantly improve outcomes (median survival was roughly 49 months post-resection) [56]. Even among patients with more advanced disease, therapeutic advances have demonstrated meaningful benefits, as prospective studies using EORTC questionnaires indicate that immunotherapy can significantly improve overall quality of life and alleviate symptoms such as dyspnea, despite the occurrence of long-term endocrine toxicities [59]. Video-assisted thoracoscopic surgery has proven to be an effective alternative to open thoracotomy in patients with NSCLC, offering reduced intraoperative blood loss, earlier chest tube removal, and shorter hospital stays [60].

Histopathology shows transitional patterns: historically, squamous-cell carcinoma (SCC) predominated (47–48%) vs. adenocarcinoma (29–38%) in heavy male smokers [41,42,61], whereas recent genomic cohorts report 70–85% adenocarcinoma in molecularly profiled cases [62,63,64,65,66,67]. In advanced Romanian cohorts, the histopathological subtype strongly predicts the first site of metastasis at diagnosis; adenocarcinomas exhibit a marked propensity for early brain and bone dissemination, whereas SCCs more frequently exhibit locoregional spread to the pleura [57], though a propensity for brain dissemination in elderly SCC patients has also been reported in a different cohort [68].

Management increasingly requires individualized multimodal strategies due to rising multiple primary malignancies (e.g., synchronous/metachronous lung with prostate/colorectal cancer), necessitating coordinated tumor board decisions and advanced targeted/immunotherapies [53,69]. In metastatic NSCLC, maintaining optimal chemotherapy dose intensity remains critical for survival even in the era of targeted therapy and immunotherapy [52].

Recent next-generation sequencing (NGS) and polymerase chain reaction studies have mapped the molecular characteristics of Romanian NSCLC patients, showing alignment with Western populations but highlighting specific regional frequencies. KRAS is the most frequent mutation, present in 29.1% of cases, with the highly actionable p.G12C variant accounting for 10.3% of the total cohort [62,70]. EGFR mutation is found in 9.3% to 14.3% of patients. It is almost exclusively found in adenocarcinomas and is significantly more prevalent among women and non-smokers. The most common alterations are exon 19 deletions (3.5%) and L858R substitutions (2.9%) [62,71,72]. Upfront NGS shows high TP53 rates alongside actionable EGFR/KRAS variants [66].

Gene fusions are rare but critical: In a cohort of 721 patients, 3.88% harbored fusions: ALK was the most frequent (1.66–1.77%), followed by RET (1.11%) and ROS1 (0.7%) [57,62,63,73]. Other reported alterations include BRAF (5.3%) and FGFR1/FGFR3 amplifications [62,74,75]. In north-western Romania, ALK overexpression exceeds 5% (in younger, non-smoking females) [73]; in northern Romania, ERCC2/XPD polymorphisms increase adenocarcinoma risk in smokers [74].

Molecular subtype influences metastasis: EGFR and KRAS are related to brain and liver metastasis, respectively [57]. In metastatic NSCLC, endocrine immune-related adverse events (e.g., thyroiditis) correlate with improved survival during immunotherapy [33].

Identifying robust prognostic markers remains a priority, as circulating protein levels at diagnosis have shown varied utility in predicting survival across different smoking phenotypes [76].

Despite advances in molecular characterization, important gaps persist in access and implementation. In north-east Romania, elderly patients were often excluded from reflex EGFR testing [42]. In real-world Romanian oncology practice, access to comprehensive molecular biomarker panels is heavily constrained by a lack of national reimbursement, often forcing reliance on pharmaceutical company-sponsored vouchers for basic EGFR, ALK, and PD-L1 testing [49].

Although most advanced tumors harbor actionable alterations (e.g., TP53, KRAS), access to matched therapies is constrained [65]. Programs like EPROPA improve genomic testing and treatment access in CEE [64]. Beyond standard genomic profiling, preclinical studies in Romania are actively exploring the in vitro antiproliferative and pro-apoptotic properties of local botanical extracts, such as Populus nigra L. buds, against human lung cancer cell lines [75].

Reported associations between ALK mutations and impaired emotional functioning, including higher anxiety levels, support integrating molecular profiling with emotional and functional assessments in routine care to inform personalized rehabilitation and supportive strategies that improve quality of life for lung cancer patients [77].

#### 3.1.3. Risk Factors and Confounders

Smoking is the undisputed primary driver. Nearly 30.7% of Romanians over age 15 (over 5.5 million people) use tobacco (40.4% in men, 21.7% in women) [40,41], with high-risk phenotypes (“rural extreme smokers”) frequently presenting with associated chronic obstructive pulmonary disease (COPD) and lung cancer [43,50,53,54,78,79]. Comorbidities like diabetes mellitus worsen adherence, quality of life, and survival, contributing to systemic inflammation (high C-reactive protein and LDH levels) and other lifestyle choices such as dietary habits, physical inactivity, and persistent alcohol consumption further negatively impact treatment outcomes [50,54,79].

Environmental and infectious factors complicate risk and diagnosis. High TB incidence causes radiologic overlap (granulomas, tuberculomas) [31,80]. Residential radon >140 Bq/m^3^ increases lung cancer risk 2.6-fold and accounts for up to 16% of deaths in non-smokers in endemic areas (e.g., Stei) [81,82]. Comparable ecological investigations in Poland, despite often limited sample sizes, have similarly reported elevated indoor radon levels among patients diagnosed with adenocarcinoma and EGFR-mutant tumors [83]. Additional risks include asbestos exposure (northern Romania) [84] and elevated polycyclic aromatic hydrocarbons in urban patients (Moldavia) [75,85,86].

Clinical outcomes are further affected by host and external factors. During the COVID-19 pandemic, Romanian lung cancer patients with severe COVID-19 more frequently developed hematological abnormalities, including lymphopenia and thrombocytopenia, which were associated with poorer overall survival [46]. Chronic inflammatory diseases (e.g., psoriasis) increase lung cancer risk [78], and high systemic inflammation combined with underweight status predicts poorer progression-free survival during immunotherapy [87].

### 3.2. Poland

#### 3.2.1. Epidemiology and Demographics

Lung cancer is the most common malignancy and the leading cause of cancer death in Poland (>22,000 deaths/year), accounting for 27.4% of male and 17.9% of female cancer mortality in 2019 [1,9,88,89,90,91,92]. Poland remains among the highest in EU lung cancer mortality [7]. Longitudinal data comparing the Greater Poland region to European averages between 1999 and 2016 demonstrate a massive 54% increase in new overall cancer cases, with lung cancer firmly holding its place as the top killer [93]. The COVID-19 pandemic severely disrupted this landscape, creating a ‘COVID-19 debt’. During 2020, there was a drastic reduction (up to 15% nationwide, and up to 38% in repurposed hospitals) in new lung cancer diagnoses, leading to a subsequent rebound in advanced-stage presentations in the post-pandemic years [45,94,95].

The epidemiological data reveals a stark gender-based divergence in recent decades. Male incidence and mortality have declined since the 1990s, paralleling reduced smoking (from ~65% in the 1980s to ~28–32% in recent years) [4,93,96,97,98,99,100,101,102], whereas female rates have increased due to cohort effects (women born 1940–1960); since 2004/2007, lung cancer has surpassed breast cancer as the leading cause of cancer death in women [8,24,89,97,103,104,105,106,107,108]. Incidence rises sharply with age, with roughly 70% of cases occurring in men over 60 years of age [99], and a notable increase among elderly women [106,109].

Regional analyses, such as those from the Świętokrzyskie Voivodeship, confirm these divergent national trends, showing a significant long-term decrease in male lung cancer incidence alongside a pronounced increase in female cases over recent decades [101], consistent with broader CEE/EU patterns of declining male but rising female mortality [9,110].

These trends align with epidemiological models demonstrating a roughly 40-year lag between peaks in smoking prevalence and lung cancer mortality [4]. Simulation forecasts further indicate that meaningful mortality reductions in Poland depend on sustained declines in smoking prevalence among younger cohorts [98].

Despite therapeutic advances, Poland retains among the lowest 5-year cancer survival rates in Europe, largely due to late-stage lung cancer [111,112]. Notably, however, women undergoing complete NSCLC resection show better long-term survival and fewer complications than men [102]. Furthermore, synchronous tumors present at older ages than metachronous cases, adding further complexity to diagnosis and management [113]. The COVID-19 pandemic also worsened outcomes by delaying diagnostics, increasing advanced-stage presentations [94].

Lung cancer distribution in Poland is markedly heterogeneous, reflecting historical and socioeconomic determinants. Mortality is highest in the northern and western regions (“Recovered Territories”), where post-1945 migrant populations exhibit higher smoking prevalence than more stable eastern communities [114]. In Greater Poland, lung cancer accounts for approximately 30% of male cancer deaths, driven by localized smoking patterns [115].

Studies show that premature mortality from lung cancer among women is historically higher in urban areas than in rural ones, reflecting higher urban female smoking rates (23.5% vs. 16.8%). However, rural populations often face lower socioeconomic status (SES), which generally correlates inversely with lung cancer survival [106,115,116,117,118,119,120,121,122,123,124]. Educational inequalities have widened, with disproportionate mortality increases among poorly educated individuals [123]. Ecological data link neighborhood poverty and social isolation to increased lung cancer risk [122].

Regional geostatistical analyses in the Opole Province demonstrate a positive correlation between unemployment and male lung cancer incidence, reflecting behavior-mediated risk (e.g., increased smoking) [118]. In low-life expectancy regions such as Lodz, lung cancer contributes substantially to years of productive life lost, particularly in men [104].

In addition to socioeconomic determinants, housing characteristics also shape environmental risk. Socioeconomic and residential factors significantly influence indoor radon concentrations, emphasizing the need for targeted home ventilation interventions to reduce modifiable carcinogenic exposures among co-habitants [119].

#### 3.2.2. Clinical, Histopathological, and Molecular Features

Polish lung cancer is dominated by late diagnosis (stage III–IV) and poor outcomes (5-year survival of roughly 13–14%) [19,45,102,125,126,127,128,129,130,131,132,133,134,135,136,137,138,139,140,141]. Consistent with changing demographics, adenocarcinoma is increasingly prevalent, particularly among women, though SCC remains a substantial burden strongly tied to the older male smoking population [102,142]. The frequency of EGFR-activating mutations in Polish patients is approximately 10.5% to 11.8%, with exon 19 deletions and L858R substitutions being the most common, predominantly seen in females and never-smokers [139,143]. Additional actionable targets such as KRAS mutations (notably the p.G12C variant) and ALK rearrangements are systematically evaluated in clinical practice [70,88]. While the application of comprehensive NGS is expanding, barriers related to testing reimbursement and turnaround times persist [144,145].

Poor ECOG performance status and advanced stage remain the strongest independent predictors of survival in highly aggressive lung cancers in the region [146]. The COVID-19 pandemic worsened this profile in Poland, with patients presenting at later stages, poorer performance status, and higher pre-treatment mortality compared to pre-pandemic periods [45].

Comorbidity burden is high, particularly COPD and cardiovascular disease (CVD). Screening cohorts in Gdańsk (MOLTEST-BIS) revealed that nearly 20% of lung cancer screening participants had COPD, and crucially, over 86% of these COPD cases were previously undiagnosed. Patients who develop lung cancer exhibit a significantly higher prevalence of CVD and COPD compared to healthy smokers [44,113,124,147,148,149,150,151,152,153]. In Polish cohorts, coexisting cardiovascular disease in advanced lung cancer substantially increases cumulative mortality risk, strongly influenced by functional status, cachexia, and heart failure severity [153]. Preventive initiatives in rural counties such as Proszowice have also revealed widespread underdiagnosis of COPD, with a significant proportion of screened individuals showing airflow obstruction closely linked to active smoking [124].

Late-stage predominance necessitates intensive palliative and multimodal care. Advanced radiotherapy techniques, including stereotactic body radiation therapy (SBRT), stereotactic radiosurgery, and volumetric modulated arc therapy, are widely used, requiring optimized dosimetry and toxicity management (e.g., dermatologic support) [126,134,135,154]. SBRT improves control and quality of life in inoperable early-stage disease [140], prophylactic cranial irradiation is generally well tolerated, particularly with application in small-cell lung cancer (SCLC) [155,156], while oligometastatic cases benefit from combined local ablative and systemic therapy (increased progression-free survival) [127]. Brain metastases are frequent and prognostically adverse, requiring blood–brain barrier-penetrant therapies and multimodal approaches (stereotactic radiosurgery, whole-brain radiotherapy, or surgery) [136,145,157]. Particular attention should be given to patients with poor performance status, specifically those classified as Radiation Therapy Oncology Group Recursive Partitioning Analysis class 3, in whom whole-brain radiotherapy has not been shown to meaningfully reduce symptom burden [158].

Postoperative outcomes in Poland are strongly shaped by comorbidities, as men undergoing lung resection exhibit higher rates of CVD and COPD [102]. Accurate pathological staging depends on adequate lymphadenectomy, with removal of more than six lymph nodes improving N classification precision and survival estimation; updated TNM criteria have also led to re-staging of advanced tumors, influencing eligibility for radical surgery [130,131]. Segmentectomy is shown to be an oncologically sound alternative to lobectomy in carefully selected stage I patients, whereas wedge resection appears associated with compromised long-term survival [159].

Postoperative high-dose-rate endobronchial brachytherapy has been shown to improve overall survival in patients with localized disease; however, only a limited proportion of patients are suitable candidates for this approach [160].

Prognosis is also influenced by immune markers: MHC II expression on tumor-infiltrating lymphocytes correlates with improved survival [132], and tumor galectin-9 is correlated with overall survival increase compared to lymphocytic galectin-9, which is correlated with increased recurrence risk [161]. Routine clinical parameters (hemoglobin, performance status, and lymphocyte to neutrophil ratio) serve as strong independent prognostic factors for overall survival, regardless of the chosen systemic treatment [138]. Higher body iron content at diagnosis correlates with increased incidence but also longer overall survival, likely reflecting improved treatment tolerance [162].

As survival from other cancers improves, the prevalence of multiple primary malignancies is increasing in Poland, with prior neoplastic disease or chemotherapy constituting significant risk factors for subsequent lung tumors [92]. Retrospective hospital analyses show that lung cancer frequently emerges as the second malignancy, strongly associated with active or former smoking [113]. Notably, patients who develop lung cancer as a subsequent tumor may demonstrate longer overall survival, possibly due to closer surveillance after their initial diagnosis [107].

Certain high-risk clinical subgroups in Poland demonstrate increased lung cancer susceptibility, including HIV-infected adults, in whom lung cancer is a leading non-AIDS-defining malignancy [150], and renal transplant recipients under long-term immunosuppression, who face a markedly elevated risk of de novo malignancies, including lung cancer [151].

Interestingly, during the COVID-19 pandemic, Polish oncology patients with solid tumors, like lung and breast cancer, demonstrated a uniquely heightened SARS-CoV-2 humoral response compared to non-cancer controls, suggesting complex interactions between systemic inflammation and anti-viral immunity [44].

#### 3.2.3. Risk Factors: Smoking, Air Pollution, and Metabolic Factors

Tobacco smoking remains the dominant risk factor for lung cancer in Poland, accounting for approximately 80–90% of cases in men and 60–70% in women [14,98,100,107,163,164]. Changing smoking patterns, where male smoking has declined but female smoking has increased, ensure that tobacco remains the primary driver behind the rising female lung cancer mortality observed in recent years [89]. Conventional cigarettes remain the primary risk factor for lung cancer, but the growing use of alternative tobacco products and e-cigarettes in Poland requires ongoing monitoring to clarify long-term carcinogenic risks [100]. Smoking and alcohol exert dose-dependent effects on disease progression and mortality [12], while cessation significantly improves overall survival [107]. Beyond direct tobacco exposure, broader lifestyle factors (including poor diet, physical inactivity, and obesity) contribute to the overall cancer burden in Poland [111].

Air pollution is a major independent driver. Poland hosts 36 out of the 50 most polluted EU cities, with high particulate matter (PM) 10, PM2.5, and benzo(a)pyrene from coal combustion and traffic [86,95,165,166,167,168,169,170,171]. PM2.5 strongly correlates with lung/bronchus cancer incidence [171], and long-term exposure to PM-bound polycyclic aromatic hydrocarbons and heavy metals increases lifetime cancer risk, especially in industrial regions like Upper Silesia and Wroclaw [169]. Research translating Polish air pollution levels into cigarette equivalents estimates that every inhabitant of Poland passively “smokes” roughly half a pack of cigarettes daily due to ambient air pollution, independently driving lung cancer morbidity [83,165,169,171,172,173,174,175,176,177,178,179,180]. Elevated PM, nitrogen dioxide (NO2), and sulfur dioxide (SO2) are linked to SCC incidence [168,181,182,183]. Seasonal “low emissions” (domestic heating) drive extreme smog and benzo(a)pyrene peaks, particularly in Silesia during cold, low-wind periods [168,170].

Occupational exposures further increase lung cancer risk in Poland, with welding fumes independently elevating risk [86] and historical asbestos processing continuing to generate cases decades after cessation [179], while newer cohorts in rubber manufacturing show no consistent excess mortality [173]. Approximately 58,000 individuals were occupationally exposed to asbestos fibers between 1940 and 1998, and considering the long latency of asbestos-related diseases, often around 30 years, the clinical consequences are likely to become increasingly apparent over time [184]. Environmental factors may also contribute: elevated mercury and copper concentrations have been detected in tumors from industrial regions [167,182], and residential radon in Eastern Poland has been investigated as an additional risk factor, often alongside occupational exposures [83]. Although ecological analyses suggest complex interactions with background radiation, pollution and tobacco remain the primary established drivers [174].

Dietary and metabolic factors modulate risk in Polish cohorts. Mediterranean-type diets are protective [175,177], whereas low vegetable intake and high glycemic/fried foods increase risk [180]. Low serum selenium associates with up to 10-fold higher risk [176,185], while certain profiles (e.g., high folate in heavy smokers) may increase risk [178]. Physical activity exerts a protective effect against lung cancer development [152].

Malnutrition is a frequent and clinically relevant issue in NSCLC. In a Polish cohort of 180 patients, 51.1% were undernourished and 23.9% were at risk of malnutrition, with nutritional impairment significantly associated with poorer quality of life across EORTC QLQ-C30 and QLQ-LC13 domains. Multivariate analysis identified malnutrition as an independent determinant of reduced physical functioning [186]. These data highlight nutritional status as a modifiable factor influencing symptom burden and quality of life, supporting its systematic assessment within comprehensive oncologic care.

#### 3.2.4. Screening Innovations and Molecular Diagnostics

To combat late-stage diagnoses, Poland has been at the forefront of implementing LDCT screening in Europe. Programs like the Pomeranian Lung Cancer Screening Program and MOLTEST-BIS have successfully screened thousands of high-risk individuals [34,187]. Polish researchers advocate for utilizing advanced mathematical risk models (like the PLCOm2012 calculator) over standard age/smoking criteria to better target the population and reduce false positives [34,142,188]. Earlier trials like the Cracow screening trial using NELSON-based volumetry, established effective nodule-detection frameworks [142].

NSCLC accounts for over 80% of cases. In the Polish population, activating EGFR mutations (which qualify patients for targeted tyrosine kinase inhibitor therapies) are found in approximately 10.5% of NSCLC patients, appearing more frequently in women and adenocarcinomas [139,143,145,189,190,191,192]. The Polish National Cancer Network (NCN) pilot programs are actively trying to improve the rates of molecular and genetic testing, which are vital for modern targeted treatments [19,144,161,193,194,195,196,197,198,199,200,201,202,203,204,205,206,207].

EGFR-positive NSCLC presents more often at stage IV and with bone metastases [139], and a strong association between EGFR mutations, adenocarcinoma histology, and female sex has previously been reported [208]. Molecular testing is high in advanced non-squamous NSCLC but underutilized in SCC [144].

For non-squamous NSCLC in Poland, determining molecular status before systemic chemotherapy is essential, as EGFR mutations and ALK/ROS1 rearrangements guide the use of highly effective targeted therapies [199,209]. Routine predictive testing, including immunohistochemistry or fluorescence in situ hybridization for ALK rearrangements, is now mandatory in adenocarcinoma workflows and directs sequential use of next-generation ALK inhibitors [200], with multinational trials involving Polish centers confirming substantial responses to agents such as crizotinib even in rare ALK-driven tumors such as inflammatory myofibroblastic tumors [207]. Identification of activating EGFR mutations established eligibility for tyrosine kinase inhibitors like erlotinib through early expanded access programs demonstrating safety and progression-free survival benefit [191,195], while real-world data later confirmed the efficacy of first- and second-generation inhibitors such as afatinib, despite overall survival sometimes trailing trial outcomes [189,192]. More recently, third-generation agents, including osimertinib, have shown significant overall survival improvement in patients with T790M resistance mutations [194].

Molecular profiling in Poland identifies rare actionable alterations such as MET exon 14, skipping mutations with potential durable immunotherapy responses [202]. Immune analyses show that galectin-9 and TIM-3 expression in NSCLC correlates with PD-1/PD-L1 signaling and poorer survival [161,210], while SCLC demonstrates distinct immune evasion marked by absent MHC Class II expression [132]. Expanding immunotherapy use necessitates testing for resistance-associated mutations such as STK11 and KEAP1 [204]. Elevated tesmin and MCM5/MCM7 expression correlate with proliferation and prognosis [193], and integrated molecular panels may outperform TNM staging in survival prediction [198]. Germline variants (MITF E318K/V320I, ATM) have minimal impact on lung cancer susceptibility in Poland [190,197]. Upfront NGS is more cost-effective compared to sequential testing and improves the detection of rare targets [201].

A preliminary Polish cohort study demonstrated that the ABCB1 C3435T gene polymorphism, particularly the TT genotype and T allele, is significantly more frequent in patients with NSCLC, suggesting an association with increased lung cancer risk [211].

Killer immunoglobulin-like receptor (KIR) genes and HLA-C variants were evaluated for their association with NSCLC susceptibility, treatment response, and clinical outcomes. KIR genotypes alone were not linked to disease risk, whereas HLA-C C1C2 heterozygosity appeared to confer a protective effect. Notably, the combined KIR2DL2+/KIR2DS2+ and HLA-C C1C1 genotype was associated with improved treatment response and significantly prolonged survival (median 23 vs. 10 months), highlighting the potential prognostic and predictive relevance of specific KIR–HLA interactions in NSCLC [212].

### 3.3. Hungary

#### 3.3.1. Epidemiology and Demographics

Hungary has long been reported by international databases such as GLOBOCAN as having the highest lung cancer incidence and mortality rates globally. However, recent analyses of the National Health Insurance Fund (NHIF) and cleaned National Cancer Registry data indicate that these figures were overestimated, largely due to mortality-based modeling and Hungary’s high autopsy rate of 35–37%, which captures many post-mortem diagnoses not recorded elsewhere in Europe [22,213,214,215]. Revised data for 2018 established a corrected incidence of 9519 cases (92.9 per 100,000 for males and 50.6 for females), placing Hungary high on the European list, but closer to the Central European average than previously assumed [213,216,217,218]. The COVID-19 pandemic caused a severe disruption, resulting in a 14.4% drop in new lung cancer diagnoses in 2020, particularly among the elderly (65+ years) and in deprived districts, leaving many cases hidden [219,220].

Hungary shows marked gender divergence in lung cancer trends, reflecting historical smoking patterns. Incidence and mortality among men have declined steadily over the past decade, approximately 2.2–2.3% annually, paralleling reduced smoking prevalence, with peak incidence in the 70–79 age group [22,217,220,221,222,223,224]. In contrast, incidence among women has increased, particularly in those aged 60–79, driven by the later uptake of smoking beginning in the 1960s [22,220,222,224,225]. A minor association links male birth in late spring with slightly reduced mortality risk [222].

Data from 2004 to 2012 showed that lung cancer ranked higher than breast, colorectal and prostate cancer in terms of median time to treatment after initial suspicion (50 days for lung cancer vs. under 30 for each of the other three), with 56.1% of patients not having received treatment during the first month after diagnosis [226], which might signal systemic delays in the care pathway.

NHIF data indicate fewer early-stage resectable NSCLC cases and a shift toward older surgical patients (66–75 age range) [221]. Population aging is expected to substantially increase lung cancer incidence and mortality, particularly in the 65–85 age group [217].

Lung cancer in Hungary shows pronounced geographic and socioeconomic disparities. Highest incidences/mortality occur in northern Hungary and the northern Great Plain (comparable to Balkan regions), while western Transdanubia reports the lowest rates (closer to Western Europe) [227,228].

#### 3.3.2. Clinical, Histopathological and Molecular Features

Roughly 75% of patients are diagnosed at stage III–IV, limiting curative options [213]. Overall, the 5-year survival rate remains poor, estimated between 14.8% and 17.9% depending on the specific study cohort, with a median survival of roughly 9 months for women and 6 months for men [6,23,213,214,223,229,230].

Adenocarcinoma has become the dominant histological subtype, accounting for roughly 42% of cases, heavily surpassing SCC (approx. 20.6%) and SCLC (10.3%). Adenocarcinoma is particularly prevalent among the rising female patient demographic [213,231]. The prevalence of classic EGFR mutations in the Hungarian population appears to be slightly lower than the broader Caucasian average, occurring in approximately 9.86% of adenocarcinomas [232]. Diagnostic algorithms emphasize the sequential or concurrent testing of KRAS, EGFR, and ALK to optimize targeted therapy allocation, given the mutually exclusive nature of these driver mutations [233,234].

However, recent data highlights a measurable improvement. Between 2011 and 2016, 5-year survival improved by 5.3% [229,235], with age-standardized 5-year net survival improving from 20.10% to 23.55% between 2011 and 2019 [235]. The COVID-19 pandemic induced a significant disruption in this landscape. In 2020, the risk of lung cancer diagnosis dropped dramatically (risk ratio of 0.87 for women and 0.91 for men compared to 2019), meaning nearly 900 fewer patients were diagnosed [220].

Modern systemic therapies and immunotherapy have nearly doubled 3-year survival in advanced non-squamous NSCLC [221,236,237]. Among early-stage resectable patients, the utilization of adjuvant therapies is steadily increasing, and the administration of targeted treatments like EGFR tyrosine kinase inhibitors has demonstrated a significant improvement in overall survival [221]. Multicenter clinical trials including Hungarian cohorts further confirm that first-line targeted therapies like erlotinib are highly effective and safe for Caucasian patients harboring activating EGFR mutations [232,237].

Despite these incremental survival gains, early mortality remains high (roughly 41.7% of patients die within 6 months from diagnosis) [23], and overall survival improvements lag behind other cancers [223]. Mortality shows seasonal peaks (November–January), likely linked to respiratory infections [214].

#### 3.3.3. Risk Factors and Confounders

In Hungary, high lung cancer incidence and mortality correlate with lower education, lower income, and higher smoking prevalence among men [227]. Homeless individuals bear a disproportionate burden, with a prevalence of 1.97% compared to 0.69% in the general population, markedly poorer survival, and 47% lower healthcare utilization costs, highlighting major access gaps [238]. Spatial analyses further demonstrate significant municipal disparities between incidence and mortality, indicating substantial unmet needs in prevention, screening, and treatment among deprived populations [228].

A critical issue within the Hungarian landscape is the phenomenon of “missed” diagnoses. The HULC study revealed that 14.24% of all lung cancer-related deaths in Hungary are diagnosed only post-mortem [215]. These patients generally utilize healthcare less frequently while alive, driven by low health literacy, fear of cancer diagnosis, and medical distrust [215,239,240].

Systemic delays are substantial, with a median of 64.5 days from suspicious imaging to first-line therapy, increasing over time [239]. Screening uptake is also behaviorally influenced. Prior mandatory TB X-ray programs reduced participation in modern LDCT among older cohorts, while smokers show higher willingness but require tailored communication to sustain compliance [240].

### 3.4. Bulgaria

#### 3.4.1. Epidemiology and Demographics

Lung cancer is a major public health issue in Bulgaria, with an average annual incidence of 43.5 per 100,000 population [241], ranking third in overall incidence (12.1% of cancers) and first in cancer mortality, accounting for 18.3% of cancer-related deaths [11,16]. It disproportionately affects men, representing 19.3% of new male cancer cases and 27.5% of male cancer deaths [242], with markedly higher crude incidence and mortality rates compared to women [243]. While male incidence in urban centers such as Sofia appears to be stabilizing, rates among women are rising significantly, reflecting shifts in tobacco use patterns [244]. While incidence rates showed a stabilizing downward trend in the last decade, the COVID-19 pandemic severely restricted access to outpatient diagnostic services, causing a marked artificial decline in newly registered cases during 2020–2021 [241].

#### 3.4.2. Clinical, Histopathological, and Molecular Features

NSCLC is the dominant histological subtype, accounting for roughly 64% of cases, while SCLC accounts for about 13% [16]. Within NSCLC, some cohorts report a very high prevalence of SCC, which strongly ties back to the heavy smoking habits of the male population [242,245].

A critical and severe feature of Bulgarian lung cancer cases is the advanced stage at diagnosis. Real-world data spanning from 2020 to 2022 showed that 61% of newly diagnosed patients presented with stage IV disease [16]. Older cohorts indicated that up to 86.3% of patients had stage IIIB or IV disease upon admission to medical oncology departments [243].

EGFR mutations occur in approximately 9.4% of NSCLC patients and are more common in women, never-smokers, and adenocarcinoma histology. Targeted therapy (e.g., gefitinib, osimertinib) is effective, emphasizing the need for broader molecular testing [245,246].

#### 3.4.3. Risk Factors

Smoking remains the primary driver of lung cancer in Bulgaria, which has a considerably higher overall smoking prevalence (approx. 37.2%) compared to many Western European countries [244]. National statistics indicate that 40% of men and 20% of women are heavy smokers, with an additional 10% in both sexes classified as occasional smokers [243].

Environmental risk is significant. In Eleshnitza, a former uranium mining area, indoor radon levels are markedly elevated (465 vs. 78 Bq/m^3^ in the rest of the district), correlating with increased lung cancer incidence [247]. National data (2013–2022) show marked inter-regional variation in lung cancer incidence, which correlates positively with residential indoor radon concentrations, especially in areas where dwellings exceed 200–300 Bq/m^3^ [241].

Molecular findings indicate a possible co-factor role of HPV types 16 or 18, with unusually high prevalence in Bulgarian lung carcinoma tissues compared to non-cancerous lung tissue and other European populations [242].

Bulgaria lacks a national lung cancer screening program, contributing, along with low public awareness and a limited radiology workforce, to predominantly late or metastatic diagnoses [16,245].

The patient pathway in Bulgaria is often prolonged, with nearly 80% of patients waiting more than two months from symptom onset to diagnosis and a median 42-day delay from diagnosis to initiation of first-line systemic therapy [16,243]. In a head-to-head comparison with Romania and Serbia, Bulgaria had the longest wait time from diagnosis to biomarker test results [248], thus highlighting a potential contributor to treatment delays.

Epidemiological surveillance is further limited by inadequate management of the National Cancer Registry since 2013, necessitating reliance on alternative real-world databases, such as the Danny platform, to identify access gaps and regional disparities in oncology services [16].

## 4. Systemic Barriers and Program Structure Optimization

### 4.1. Overcoming Structural and Socioeconomic Barriers

#### 4.1.1. Structural and Workflow Barriers

A major CEE barrier is fragmented, non-interoperable registries, which limit the ability to track screening and follow-up. In Romania, the current landscape is characterized by a lack of a comprehensive national lung cancer registry, forcing reliance on fragmented regional data that limits the ability to monitor incidence trends or formulate robust health policies [3,20,40,49,64,65,69,72,144,145,200,239,249]. Bulgaria’s National Cancer Registry has been unreliable since 2013 [16]. Poland, via the National Health Fund (NFZ) and NCN, has better infrastructure but still faces data gaps and reporting errors [250], with persistent primary care delays despite the implementation of “fast-track” oncological cards (DiLO), which reduce hospital stays when compared to emergency routes [19,94,129,146,195,251,252,253].

Across CEE, limited epidemiological research impairs cancer control planning and sustains survival gaps compared to older EU states [3,10]. In Hungary, diagnoses shift toward more advanced stages over time [223], while in Poland, rapid diagnosis in SCLC reflects late symptomatic presentation [146].

Primary care physicians serve as a critical but variable filter in the regional screening and diagnostic pathway. A comparative vignette study across five Balkan countries showed that PCPs in Romania and Bulgaria were more likely to refer patients with suspicious symptoms to specialists than those in Slovenia and Croatia [254]. However, this referral tendency is constrained by limited direct access to advanced imaging, as fewer than a quarter of PCPs in Bulgaria and Romania can directly order CT or MRI scans [254]. This disconnect creates a “referral paradox” where high suspicion indices at the primary care level meet structural roadblocks, delaying the confirmatory diagnosis required for effective screening or early detection.

In Poland, prolonged diagnostic and treatment intervals and decentralized hospital networks generate undocumented delays and survival bias [105,253]. Atypical symptoms such as abdominal pain can further delay diagnosis [251].

Systemic inefficiencies in molecular testing persist across CEE countries, where many patients progressing on first-line therapy are not tested for resistance mutations such as T790M and experience high attrition before second-line treatment [72]. In the Polish REFLECT cohort, nearly one-third of patients progressing on first-line EGFR inhibitors were not tested for T790M, limiting access to subsequent targeted therapies [145]. Earlier use of NGS could mitigate rapid clinical decline that often precludes late-stage patients from receiving appropriate targeted treatment or trial enrollment [65]. Rare targets require centralized pathology and international collaboration, yet access to biomarker-driven trials remains limited [64,207].

Treatment delivery across CEE remains inconsistent, with historically suboptimal systemic therapy use in advanced NSCLC in countries such as Poland and Hungary [20] and underutilization of key staging procedures like PET-CT and brain imaging in stage III disease [21]. Radiologist shortages and limited CT capacity further constrain both advanced care and large-scale screening implementation, underscoring the need for coordinated planning and artificial intelligence-supported workflows [30].

Targeted institutional solutions show increased benefits for CEE patients. For example, Poznań centers improved pathways via urgent PET-CT and multidisciplinary boards (recommending concurrent radiochemotherapy) [128], Romanian multidisciplinary teams achieved high consensus in systemic therapy [69], and Hungary implemented European Society of Thoracic Surgeons-based surgical benchmarking [249]. Additionally, the introduction of dedicated oncology-care coordinators has been highly valued by patients and has improved navigation through the diagnostic pathway [185].

The COVID-19 pandemic imposed a substantial “COVID-19 debt” on already strained systems, with northern Polish centers reporting marked declines in new lung cancer diagnoses followed by rebound surges in 2021–2022 due to service reconfigurations and patient fear [94]. These disruptions also worsened patients’ mental health, highlighting the need for integrated psycho-oncological support and oncology coordinators [255].

#### 4.1.2. Geographic and Economic Inequalities

In CEE, the “inverse care law” is evident: highest-risk populations have the poorest access to diagnostics [10]. In Romania, rural high-risk clusters (e.g., “rural heavy smokers”) present late-stage disease with severe comorbidities, compounded by centralized imaging inaccessible to remote areas [43]. In Poland, resource distribution is uneven, with fewer oncologists and hospital beds in rural voivodeships compared to Warsaw or Silesia, causing diagnostic delays and travel barriers to LDCT and specialized care [95,105,122,256,257,258]. Residence influences outcomes, with lower survival in rural and elderly populations due to limited access [116,259].

Access gaps extend to treatment. In Central Poland, rural radiotherapy availability lags despite overall expansion, limiting salvage options and worsening metastatic outcomes [105,133]. Socioeconomic vulnerability is critical. Homeless individuals in Hungary face a threefold higher lung cancer prevalence (1.97% vs. 0.69%) and worse survival, yet incur 47% lower disease-specific healthcare costs despite universal coverage, reflecting major access gaps [238]. The Hungarian OncoNetwork program shows that structured patient navigation improves timeliness of care and survival (HR 0.63) in these vulnerable populations [228,238,253,260].

Environmental exposures, including PM10-bound benzo(a)pyrene in industrial regions, further elevate lung cancer risk among socioeconomically disadvantaged populations [168], while tobacco use and diet drive regional mortality and DALY burden in Poland [93]. The higher mortality-to-incidence ratios in Eastern versus Western Europe underscore the need for equitable access and expanded LDCT screening, particularly given wide variability in smoking prevalence, healthcare spending, and productivity losses across EU states [5,261,262].

Mitigation requires targeted interventions: focus on low-education groups with high smoking burden [123], early prevention in men before peak incidence [99], and lifestyle optimization (nutritional patterns) [175]. These efforts should be accompanied by measures to increase awareness of smoking-related risk, strengthen self-efficacy for smoking cessation, and reduce perceived barriers to adopting healthier behaviors such as a balanced diet and regular physical activity [263]. Community-based programs (questionnaires, spirometry) have proven effective in rural populations, reducing access gaps [124].

#### 4.1.3. The “Fear Factor” and Medical Distrust

Barriers to lung cancer screening in CEE span patient fear or stigma, provider knowledge gaps, and logistical constraints. In Hungary, the HULC study shows a substantial proportion of cases diagnosed post-mortem, reflecting a failure to engage patients while they were alive [215]. In Poland, smokers demonstrate lower awareness of tobacco-related risks compared to non-smokers, with disadvantaged groups showing poor knowledge of active/passive smoking harms, limiting cessation and screening uptake [121,164].

Missed diagnoses are largely driven by reluctance to seek care due to low health literacy and fear of diagnosis [31], compounded by smoking-related stigma and medical distrust [28,79,80,121,164,203,255,264,265,266,267,268]. Recent data suggests that despite improvements in survival, these psychosocial barriers remain a critical “invisible” hurdle to reducing mortality in the region [220]. The COVID-19 pandemic further worsened these effects in Poland, increasing depression, anxiety, and post-traumatic stress disorder, and reducing illness acceptance [255].

Diagnostic and treatment processes add further burden: invasive procedures (e.g., bronchoscopy) in Romania induce anxiety or depression and reduce follow-up adherence [80], while chemotherapy-related toxicity worsens psychological distress and impacts quality of life in Polish patients [265].

Communication gaps exacerbate these challenges. In Poland, NCN pilot surveys identified the scope and flow of information from medical staff as the lowest-rated aspect of care [203], and most male lung cancer patients report significant unmet non-medical needs, particularly for clearer prognostic communication and psycho-emotional support [267]. This is especially observed in men with lower levels of education who live in rural areas and are pensioners [269].

In the Pomeranian Lung Cancer Screening Program, participation among high-risk individuals was driven primarily by media campaigns, family influence, and cancer awareness rather than healthcare professional referral [270].

Psychological resilience modifies outcomes: higher life satisfaction and adaptive coping improve illness acceptance [268], while access to effective radical treatments can reduce anxiety and depression [140].

### 4.2. Optimizing Workflow: Risk Prediction and Biological Triage

#### 4.2.1. Enhancing Selection: The Risk-Prediction Model

Standard NCCN or US Preventive Services Task Force criteria (age, pack-years) are suboptimal in CEE. In Poland, standard NCCN Group 1 criteria may cause “over-screening,” including lower-risk individuals and straining radiology capacity [34,261,271]. Conversely, in Romania and Hungary, these smoking-centric criteria risk excluding specific high-risk subpopulations. Recent clustering analyses from western Romania (Timis County) identified distinct patient phenotypes, such as elderly never-smokers and women with adenocarcinomas, who do not fit the traditional “heavy smoker” profile, yet present with advanced disease [43]. Furthermore, the genomic landscape in Romania reveals a notable prevalence of EGFR mutations and other driver alterations (e.g., ALK, ROS1) in non-smoking or light-smoking populations, particularly women, who would be systematically invisible to screening protocols based solely on tobacco consumption [62,63,73,237]. Across the EU, tens of millions of adults meet high-risk criteria for lung cancer, underscoring the need for efficient screening to avoid overwhelming health systems [261]. In CEE, demographic anomalies and sex-specific smoking patterns require localized, multivariable risk models beyond age and pack/year criteria [222], particularly to identify never-smokers and women, who are more likely to harbor driver mutations yet often fall outside standard eligibility thresholds [139].

Additional regional predictors should be integrated: family history (high independent risk of early-onset disease) [271], indoor radon in Romania [82], and occupational exposure as highlighted in Poland’s Amiantus monitoring program for former asbestos workers [183].

While standard diameter-based LDCT screening, such as that used in the NLST, yields a high false-positive rate and a positive predictive value (PPV) of only 3.8% [272], incorporating refined measurement criteria and mathematical risk models significantly improves diagnostic efficiency. In the Hungarian HUNCHEST-II program, adopting volumetric analysis and volume doubling time (VDT) instead of standard diameter-based assessment dramatically improved the PPV to 58%, though false-positive rates (42%) still warrant the use of additional triage tools [273]. Addressing these disparities further requires moving from binary eligibility criteria (age and smoking history) to mathematical risk models. In the Polish MOLTEST-BIS program, applying the PLCOm2012 risk model (cutoff ≥1.3%) reduced the screening population by 17.6% compared to standard NCCN criteria, avoiding unnecessary scans while missing only 2.6% of detected lung cancers, achieving an area under the curve (AUC) of 0.717 [34]. Polish experts therefore support PLCOm2012 to guide shared decision-making and better target limited public health resources to those at highest risk [30,274,275,276,277]. Machine learning further improves risk classification, recurrence prediction, and patient trajectory modeling [276,277].

Alternative and complementary strategies include digital chest tomosynthesis (Hungary) as a lower-dose, cost-effective option [278] and integration of smoking cessation within LDCT programs to enhance cost-effectiveness [27]. Finally, severe cardiac and systemic comorbidities significantly complicate patient selection for invasive interventions, demanding holistic mortality risk assessments prior to surgical triage [153].

#### 4.2.2. Biological Triage: Serum Metabolites and Genetics

Economic simulation analyses across nine countries with diverse economy sizes and various healthcare systems suggest that expanding biomarker testing is associated with improved overall survival and fewer treatment-related toxicities, especially in populations where actionable mutations are more prevalent [279]. While initial expenditures for testing and targeted therapies increase, subsequent healthcare costs, including those linked to adverse event management and end-of-life care, tend to decline [279].

To further reduce LDCT false positives and evaluate indeterminate pulmonary nodules, Polish and Italian screening programs have explored serum biomarkers as non-invasive triage tools to be used in conjunction with LDCT. Without biological adjuncts, benign granulomas heavily inflate false-positive rates. A mass spectrometry study identifying a serum peptidome signature demonstrated an impressive ability to pre-select patients and discriminate benign from malignant nodules. In the discovery cohort, the signature achieved an AUC of 0.88, 100% sensitivity, and a negative predictive value (NPV) of 100%, with a PPV of 48% (a massive improvement over LDCT alone). In an independent validation cohort, it maintained a strong NPV of 88% and an AUC of 0.73 [35].

Similarly, a gas chromatography/mass spectrometry metabolomics approach in the Gdańsk screening cohort identified a panel of nine serum metabolites (including downregulated amino acids, carboxylic acids, and tocopherols) that separated early-stage lung cancer patients from healthy matched controls with 100% sensitivity and 95% specificity [280]. Additionally, a 7-miRNA signature (including stable components like miR-122 and miR-21) yielded an AUC of up to 80% in training cohorts, though performance drops in distinct validation datasets highlight the need to adjust for histological subtypes like adenocarcinoma [36]. Although the large inter-cohort heterogeneity observed in multicenter validation studies (where overall AUCs occasionally dropped to 0.51–0.60) suggests that universal metabolic biomarkers remain challenging to scale without adjusting for lifestyle confounders [281], the consistently high NPVs (88–100%) indicate that locally calibrated liquid biopsies can act as highly effective gatekeepers. These tests can safely rule out malignancy and drastically reduce the number of patients subjected to unnecessary follow-up imaging or invasive biopsies.

Additional serum biomarkers have been explored for biological risk stratification. Lower serum folate is associated with increased lung cancer risk [282], while elevated serum iron and ferritin correlate with higher incidence [162]. Trace elements also show prognostic relevance: higher selenium and zinc levels associate with improved survival, whereas elevated copper predicts poorer outcomes [283], with regional data confirming selenium’s survival benefit in early-stage disease [185]. Genetic variants in selenoprotein genes such as GPX1, alongside trace element assessment, may further refine risk stratification in screening programs [176].

On the other hand, DLEC1, MLH1, and TUSC4 mRNA expression has limited value as a diagnostic or prognostic tool in NSCLC within the Polish population, despite their potential role in tumorigenesis [284]. Clinical markers like baseline neutrophil-to-lymphocyte ratio and body mass index, as well as circulating proteins (IL8, hepatocyte growth factor), provide prognostic value, especially in never-smokers [76,87]. In Romania, dynamic cytokines (IL-10, TNF-α) predict early therapeutic response and survival [285].

Non-invasive biological stratification is being explored through respiratory profiling. Altered bronchial microbiota, including higher prevalence of Acinetobacter spp. in malignant disease, may help differentiate cancer from benign conditions [80], while analysis of volatile organic compounds (VOCs) and exhaled breath condensate (EBC) is under investigation to improve early detection sensitivity and specificity [286].

The clinical utility of these emerging biomarkers is significantly challenged by the high regional prevalence of COPD and TB. While TB and COPD are well-recognized as major radiological confounders, their profound impact on the lung’s inflammatory and immune profile also acts as a critical confounder for biomarker performance. Because patients with COPD face a 2- to 4-fold increased risk of developing lung cancer, they represent a primary demographic in screening programs [27]. The pathogenesis of COPD involves chronic airway inflammation, structural remodeling, and significant alterations in pulmonary immune responses [204]. These inflammatory changes can severely alter the proteomic and metabolic profiles of EBC and serum. For instance, inflammatory cytokines such as TNF-α and IL-1β are often elevated in the EBC of COPD patients, which can overlap with the biomarker signatures used to detect early-stage lung cancer and reduce diagnostic specificity [286]. Similarly, active or latent TB drives systemic inflammatory responses and oxidative stress, which can skew the levels of VOCs and other metabolic biomarkers intended for cancer detection. Consequently, to minimize false-positive results, future biomarker panels must be rigorously validated to accurately differentiate between malignancy and the background molecular ‘noise’ generated by non-malignant chronic inflammation and granulomatous diseases [286].

Integration of genetic profiling into screening is supported by the distinct molecular landscape observed in Romania. NGS analyses show KRAS mutations in 29.1% of cases, with p.G12C accounting for 10.3% [62], and EGFR mutations in 9.3–14.3%, particularly exon 19 deletions and L858R substitutions, often in non-smoking women who may fall outside pack/year criteria [62,71]. This prevalence of actionable drivers supports embedding reflex molecular testing, including liquid biopsy or rapid tissue profiling at nodule detection, to enable timely triage into targeted therapies and reduce regional diagnostic delays [63,66,285,287].

Nevertheless, real-world data indicate that KRAS status alone does not independently predict response to platinum-based chemotherapy [233], underscoring that molecular alterations do not automatically translate into clinically actionable stratification tools. This reinforces the need for robust prospective validation before integrating molecular or circulating biomarkers into screening workflows.

Additional biomarkers are under evaluation. Tissue-level markers such as tesmin mRNA [193], non-coding RNAs, and protein panels, including FOXM1 and Flotillin-1 [198], and checkpoint co-expression profiles such as TIM-3 and PD-L1 in early-stage lesions [210] may refine prognostication and adjuvant decision-making. Integrated tissue and liquid biopsy strategies are feasible with rapid turnaround and expand actionable findings despite reimbursement barriers [66], while circulating tumor DNA supports dynamic monitoring and is being evaluated in multinational studies such as APPLE with central coordination in Gdańsk [205]. Hungarian data further demonstrate that machine learning analysis of serum N-glycome alterations may aid post-resection surveillance [288].

Therapeutic modifiers must also be considered, as systemic steroids and proton-pump inhibitors may attenuate immunotherapy efficacy by altering the microbiome and immune response [33]. Advances in transcriptomic and epigenetic profiling, including DNA methylation analysis in sputum or blood, hold promise for developing robust non-invasive screening markers [196].

A structured quantitative comparison clearly illustrates the necessity and value of transitioning from LDCT alone to a multimodal approach:

- LDCT alone (standard diameter-based) has a high sensitivity but very poor PPV (~3.8%), resulting in over 90% false-positive rates [272].
- LDCT + volumetry (e.g., NELSON, HUNCHEST-II) improves PPV significantly up to 58%, though overdiagnosis and false positives still pose a moderate burden (42% false positive rate) [273].
- LDCT + mathematical risk models (e.g., PLCOm2012) filter out ~17% of low-risk individuals who would normally trigger false alarms, while preserving 97.4% of actual cancer detections (AUC 0.717) [34].
- LDCT + serum biomarkers (metabolomics/miRNA) provide excellent negative predictive values (88–100% NPV, 95% specificity). When applied to indeterminate nodules found on LDCT, this step confidently excludes false positives without resorting to invasive tissue biopsies [35,280].
- By sequentially applying risk-prediction modeling for enrollment, volumetric LDCT for detection, and liquid biopsies for triage, the proposed multimodal strategy shifts from a hypothetical framework to a highly supported, evidence-based methodology that maximizes detection efficiency while minimizing systemic burden and patient harm.

Furthermore, the clinical translation of these biomarkers is heavily complicated by chronic inflammatory conditions, notably COPD, which is highly prevalent among lung cancer screening participants. Chronic lung inflammation produces a “senescence-messaging secretome” rich in interleukins, chemokines, and growth factors that overlaps significantly with cancer-induced systemic inflammation [141]. For instance, pro-inflammatory cytokines such as IL-6 and TNF-α, as well as acute-phase proteins like C-reactive protein, are elevated not only by NSCLC but also by coexisting COPD and CVD [289]. This shared inflammatory milieu can easily obscure tumor-specific signals. Similarly, serum metabolomic profiles exhibit large inter-cohort heterogeneity due to lifestyle and inflammatory confounders, markedly impairing the accuracy of classification models if not strictly adjusted for these factors [281]. The presence of smoking-induced extracellular matrix remodeling, reflected in altered levels of matrix metalloproteinases, further blurs the lines between chronic tissue damage and early carcinogenesis [290]. Thus, generic inflammatory or metabolic panels risk yielding high false-positive rates unless carefully calibrated to account for the baseline chronic inflammation inherent to heavy smokers.

#### 4.2.3. Handling the “TB Confounder”

**Figure 3:**
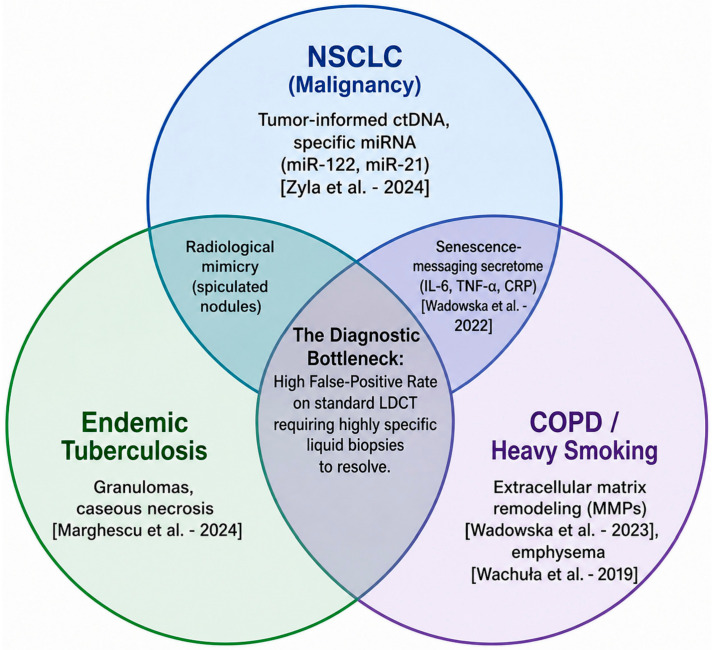
Conceptual diagram of the CEE confounder overlap. Endemic granulomatous infections (tuberculosis) and smoking-induced chronic inflammation (COPD) generate radiological and biological signals that heavily overlap with early-stage non-small-cell lung cancer (NSCLC), leading to false-positive LDCT results and reduced specificity in generic inflammatory biomarker panels [31,36,141,289,290]. ctDNA—circulating tumor DNA; miRNA—microRNA; CRP—C-reactive protein; IL-6—interleukin 6; TNF-α—tumor necrosis factor alpha; LDCT—low-dose CT; MMP—matrix metalloproteinase.

A key CEE barrier is TB-related granulomatous inflammation, a major diagnostic confounder (Figure 3). In Romania, where TB incidence is high, many resected solitary pulmonary nodules are benign (tuberculomas, fibronodular or calcified lesions) yet mimic malignancy on CT (spiculation, solid pattern) [31,291]. This overlap increases false positives, “over-surgery,” anxiety, and costs when standard criteria are applied without local adaptation [58,67,291,292].

TB confounder extends beyond radiological mimicking to significantly impair biological triage. Granulomatous inflammation, whether active or latent, alters the host’s systemic immune response by recruiting immune cells and releasing pro-inflammatory cytokines that can generate false-positive signals in blood-based biomarker assays [31,80]. Common prognostic markers, such as the neutrophil-to-lymphocyte ratio or generic acute-phase proteins, are highly sensitive to these infectious and granulomatous processes, drastically reducing their specificity for malignancy [87,243]. Consequently, in TB-endemic regions, applying unadjusted biomarker panels may paradoxically increase the false-positive rate. To overcome this, screening programs must rely on highly specific molecular signatures, such as tumor-informed circulating tumor DNA or specific microRNA panels like miR-122 and miR-21 [36], that can reliably differentiate true oncogenesis from infection-induced granulomatous inflammation.

To mitigate this "TB confounder," screening programs in the region are increasingly moving away from the diameter-based measurements used in the NLST in favor of volumetric protocols derived from the European NELSON trial [27,142,293]. The Hungarian HUNCHEST program uses volume and VDT, improving positive predictive value and reducing false positives compared to diameter-based approaches [272,273,278,294,295]. This “wait-and-scan” strategy safely excludes benign granulomas and is essential in TB-endemic settings [240,295,296].

Metabolic tumor volume on PET-CT further predicts occult mediastinal metastasis in NSCLC [293], while accurate staging requires multimodal assessment: combining CT with negative endobronchial ultrasound (EBUS) improves exclusion of mediastinal invasion [297], and endoscopic ultrasound-guided fine-needle aspiration offers a minimally invasive alternative to surgical staging for suspicious nodes or central lesions [296]. For peripheral nodules, imaging-guided transthoracic biopsy in Romanian centers achieves diagnostic success rates above 95% [67], and EBUS-guided transbronchial needle aspiration (EBUS-TBNA) with rapid on-site evaluation streamlines staging and reduces unnecessary thoracotomies [13,58].

#### 4.2.4. Economic Evaluation of Multimodal Screening

Although a formal economic evaluation is beyond the scope of this review, the existing literature strongly elucidates why a multimodal lung cancer screening strategy is highly cost-effective. Lung cancer imposes a massive economic burden, accounting for the highest costs among all cancers in the EU (approximately €18.8 billion annually, representing 15% of overall cancer costs), alongside significant indirect costs related to productivity loss from premature mortality and short-term work absence [262,298,299]. Therefore, investing in early detection is recognized as an economically sound approach.

Cost-effectiveness analyses of LDCT screening conducted in CEE settings strongly support its implementation. In Poland, a cost-effectiveness analysis determined that the incremental cost-effectiveness ratio (ICER) for LDCT screening was highly favorable at 1353 EUR per year of life gained [300]. Similarly, in Hungary, modeling of annual LDCT screening for high-risk smokers yielded an ICER of 5707 EUR/quality-adjusted life-year [218]. Recent evaluations based on the Hungarian HUNCHEST-II screening program further confirmed that both annual and biennial LDCT screening remain well below the national willingness-to-pay thresholds [2,295]. While LDCT screening alone involves substantial initial infrastructure and operational costs, studies such as the Hungarian HUNCHEST program have demonstrated that screening can become cost-saving by the tenth year due to the significantly reduced expenses of treating early-stage versus advanced-stage disease [294].

**Figure 4:**
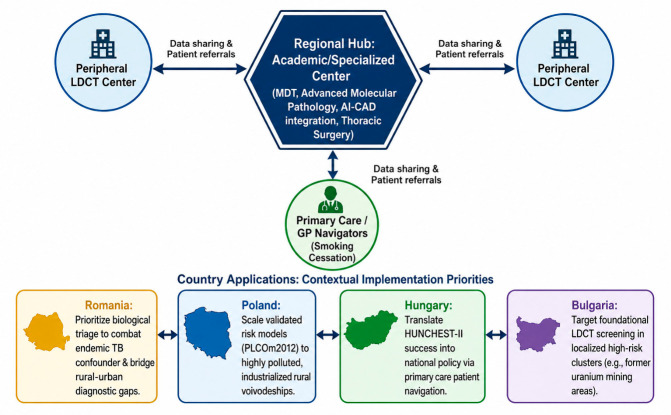
The proposed centralized “Hub-and-Spoke” infrastructure network for CEE countries, combined with national-level strategic priorities for implementation. LDCT—low-dose CT; MDT—multidisciplinary team; AI-CAD—artificial intelligence computer-aided detection; GP—general practitioner; TB—tuberculosis.

For this proposed framework to be actionable across Romania, Poland, Hungary, and Bulgaria, the infrastructure design must directly address the severe regional shortages of thoracic radiologists and specialized diagnostic equipment. A decentralized, fragmented approach is unfeasible. Instead, the implementation requires a centralized “hub-and-spoke” network model (Figure 4), as successfully piloted in Poland’s National Program of Early Lung Cancer Detection. In this model, one leading academic or highly specialized center (the “hub”) is appointed per region, working in tandem with two to four peripheral LDCT screening centers (the “spokes”) [28]. All radiological interpretation, biological triage, and critical care decisions are centralized within the hub’s multidisciplinary team (MDT), ensuring high-quality, standardized nodule management while allowing broad geographic access to the physical CT scanners [28].

**Figure 5:**
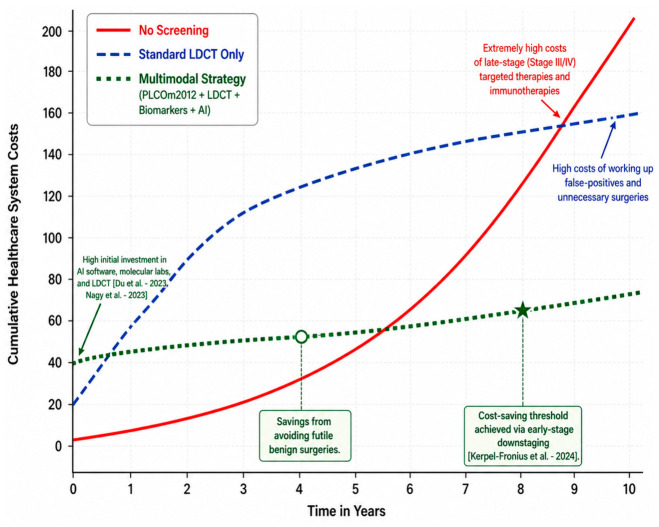
Theoretical 10-year cumulative cost trajectory of lung cancer management. While the multimodal screening strategy (LDCT + AI + Biomarkers) requires the highest initial capital investment, it crosses the cost-saving threshold sooner than standard LDCT by drastically reducing the downstream costs of futile invasive diagnostics for benign lesions and advanced-stage therapies [218,273,301]. LDCT—low-dose CT; AI—artificial intelligence.

To offset the workforce burden in these hubs, the integration of deep-learning computer-aided detection (DL-CAD) software is a mandatory infrastructural requirement, significantly reducing reading times and generating long-term operational cost savings (Figure 5) [301]. From a budgetary perspective, while initial capital investments are required for LDCT and liquid biopsy assays, health economic modeling indicates that the financial burden is highly manageable. For instance, comprehensive analyses in Hungary project suggest that a 10-year nationwide LDCT-based screening program would require only approximately 0.08% of the country’s total public healthcare spending [218]. By optimizing the pathway with risk models and biomarkers, the overarching infrastructure operates efficiently, preventing the healthcare system from being overwhelmed by false-positive interventions and making the strategy highly sustainable across CEE target systems.

Crucially, the transition from standard LDCT to the proposed multimodal strategy enhances this economic profile across several critical dimensions. First, integrating structured smoking-cessation interventions directly into the screening program is vital. While this slightly increases upfront costs, it has been shown to improve overall cost-effectiveness, as measured by quality-adjusted life years gained, by 1.7- to 5.4-fold [14,27,30,274].

Using multivariable mathematical risk calculators (e.g., PLCOm2012) instead of simple age/smoking criteria reduces the number of individuals requiring scans by nearly 18% without missing significant cancer cases [34]. This ensures that screening is restricted to the highest-risk populations, maximizing the diagnostic yield while minimizing the overall budget impact [30].

Integrating DL-CAD software can substantially reduce the reading time for radiologists, showing strong cost-saving potential by offsetting initial software investments [301]. Furthermore, combining LDCT with volumetric nodule assessment protocols (as seen in the NELSON trial) drastically reduces the false-positive rate.

A major driver of screening costs stems from the clinical management of false-positive indeterminate nodules, which often lead to expensive invasive diagnostic procedures (e.g., biopsies, mediastinoscopies) and the treatment of their subsequent complications [35,37]. While broad molecular testing and liquid biopsies require an initial financial investment, they optimize patient pathways by acting as highly specific gatekeepers [279]. By successfully differentiating between malignancy and benign confounding lesions, this strategy minimizes the profound economic burden and patient harm associated with unnecessary downstream investigations, ultimately providing better value for healthcare resources [27,29].

Ultimately, a multimodal strategy not only improves clinical outcomes but serves as a fundamental mechanism for ensuring the financial sustainability of screening programs in resource-constrained environments.

#### 4.2.5. Actionable Multimodal Pathway: Step-by-Step Thresholds

**Figure 6:**
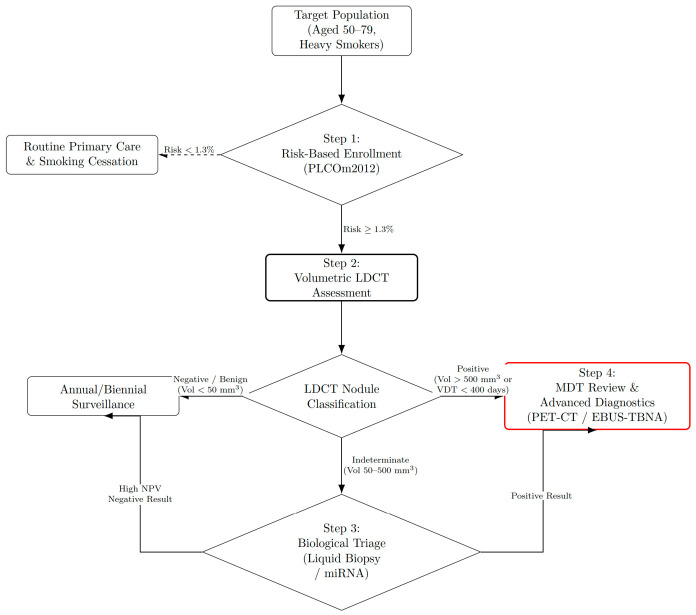
Clinical decision algorithm for the proposed multimodal lung cancer screening pathway in CEE. The framework sequentially integrates the PLCOm2012 risk model, volumetric LDCT, and biological triage to minimize false positives and invasive procedures. LDCT—low-dose CT; VDT—volume-doubling time; MDT—multidisciplinary team; EBUS-TBNA—endobronchial ultrasound-guided transbronchial needle aspiration; miRNA—micro RNA; NPV—negative predictive value.

To transition this multimodal framework from an aspirational concept to an actionable clinical pathway, screening programs in CEE must adopt rigidly defined thresholds at each step of the patient journey (Figure 6):

- Step 1: Risk-Based Enrollment. Instead of standard age and pack/year criteria, candidates are filtered using the PLCOm2012 mathematical risk-prediction model. The trigger threshold for enrollment is a ≥1.3% probability of developing lung cancer over 6 years. Validation in the Polish MOLTEST-BIS cohort demonstrated that this specific cutoff safely excludes ~17.6% of lower-risk individuals from unnecessary radiation and anxiety while capturing 97.4% of actual lung cancer cases [34].
- Step 2: Volumetric LDCT Assessment. Scans must be evaluated using volumetric analysis and VDT rather than simple linear diameter [273]. Based on protocols validated in the Gdańsk screening programs, nodules >500 mm^3^ or with a VDT of <400 days are classified as highly suspicious (positive) and immediately trigger a MDT review [275]. Nodules below these thresholds but above baseline benign dimensions are classified as indeterminate, triggering a 3-month follow-up scan or immediate biological triage.
- Step 3: Biological Triage for Indeterminate Nodules. Patients with indeterminate nodules, particularly in TB-endemic regions where granulomatous scarring mimics malignancy, undergo blood-based triage using validated serum peptidome or miRNA signatures (e.g., miR-122 and miR-21) [35,36]. An assay result yielding a high NPV (NPV threshold ≥ 88–100%) serves as the threshold to safely rule out malignancy and return the patient to routine annual/biennial surveillance, halting the cascade of invasive diagnostics.
- Step 4: Advanced Diagnostics and MDT. Patients with positive LDCT volumetry or biomarker-positive indeterminate nodules proceed to advanced staging. Guidelines mandate the use of PET-CT to exclude mediastinal invasion [37], followed by minimally invasive tissue acquisition via EBUS-TBNA or imaging-guided transthoracic needle biopsy for peripheral lesions [32,67]. Crucially, surgical resection for benign lesions must be kept strictly under a 10% threshold [275].

Consequently, while the core principles of this multimodal framework remain consistent, its practical implementation must be uniquely tailored to address the distinct epidemiological and structural realities of each national context. The primary barrier in Romania is the high prevalence of endemic tuberculosis and severe rural–urban disparities in accessing imaging [17]. Therefore, the Romanian framework must prioritize the strict integration of biological triage (liquid biopsies) to confidently exclude benign granulomatous lesions before resorting to invasive procedures [31,67]. Furthermore, implementation requires a strong hub-and-spoke model (Figure 4) to bridge the gap between rural heavy smokers and centralized molecular pathology capabilities in tertiary centers [40].

Poland possesses strong foundational expertise from regional screening pilots (e.g., MOLTEST-BIS) and pilot NCN, but faces significant geographic inequalities and air pollution burdens [169,250]. The Polish framework should focus on scaling these existing multivariable risk-prediction models (such as PLCOm2012) nationwide, expanding access beyond specialized urban centers to rural and highly polluted industrial voivodeships where SCC incidence remains disproportionately high [34,181].

Characterized by historically high lung cancer mortality and a concerningly high rate of diagnoses made only post-mortem, Hungary’s priority is engaging patients earlier in the diagnostic pathway [215,220]. The Hungarian framework must capitalize on the proven clinical effectiveness of the recent HUNCHEST-II pilot by translating it into a fully funded national program [273]. Crucially, this requires the integration of structured patient navigation programs at the primary care level to combat medical distrust and improve screening adherence among the growing demographic of female smokers [228,260].

Bulgaria currently lacks any formalized screening program and struggles with an overwhelming proportion of patients (over 60%) presenting at stage IV [16]. The Bulgarian framework must take a more foundational approach, initially targeting localized high-risk geographic clusters, such as populations exposed to high indoor radon levels near former uranium mining sites [241]. By initially combining basic LDCT screening with essential staging improvements in these specific high-risk zones, Bulgaria can gradually build the capacity needed for broader national implementation [254].

## 5. Conclusions

Lung cancer control in CEE is constrained not only by the limited scale of screening programs, but also by the mismatch between Western screening models and regional realities. Across Romania, Poland, Hungary, and Bulgaria, late-stage diagnosis persists alongside fragmented registries, diagnostic delays, workforce shortages, unequal access, medical distrust, and major confounders such as air pollution, radon, COPD, and tuberculosis-related granulomatous disease.

Current evidence supports LDCT screening for high-risk populations when embedded in a structured multimodal pathway rather than used as a stand-alone test. Risk models can refine selection, volumetric LDCT can improve nodule assessment, and selective biological triage may reduce unnecessary invasive procedures, especially in settings with frequent benign inflammatory or granulomatous lesions. However, successful implementation also depends on centralized coordination, multidisciplinary review, interoperable registries, adequate diagnostic capacity, smoking-cessation support, and patient navigation.

Overall, structured and context-adapted LDCT programs represent the most evidence-based near-term strategy for CEE, whereas biomarker-based triage remains promising but not yet ready for routine population-wide use. Future work should focus on pragmatic implementation, prospective validation, health-economic evaluation, and country-specific adaptation.

## Data Availability

The original contributions presented in this study are included in the article. Further inquiries can be directed to the corresponding author.

## Abbreviations

The following abbreviations are used in this manuscript:

AUC	Area under the curve
CEE	Central and Eastern Europe
COPD	Chronic obstructive pulmonary disease
CVD	Cardiovascular disease
DL-CAD	Deep-learning computer-aided detection
EBC	Exhaled breath condensate
EBUS	Endobronchial ultrasound
EBUS-TBNA	Endobronchial ultrasound-guided transbronchial needle aspiration
ICER	Incremental cost-effectiveness ratio
KIR	Killer immunoglobulin-like receptor
LDCT	Low-dose computed tomography
MDT	Multidisciplinary team
NCCN	National Comprehensive Cancer Network
NCN	National Cancer Network
NFZ	National Health Fund
NGS	Next-generation sequencing
NHIF	National Health Insurance Fund
NLST	National Lung Screening Trial
NPV	Negative predictive value
NSCLC	Non-small-cell lung cancer
PCP	Primary care physician
PM	Particulate matter
PPV	Positive predictive value
SBRT	Stereotactic body radiation therapy
SCC	Squamous-cell carcinoma
SCLC	Small-cell carcinoma
SES	Socioeconomic status
TB	Tuberculosis
VDT	Volume doubling time
VMAT	Volumetric arc-therapy
VOC	Volatile organic compound
